# Correction to: Emerging models and trends in mental health crisis care in England: a national investigation of crisis care systems

**DOI:** 10.1186/s12913-021-07295-2

**Published:** 2021-12-09

**Authors:** Christian Dalton-Locke, Sonia Johnson, Jasmine Harju-Seppänen, Natasha Lyons, Luke Sheridan Rains, Ruth Stuart, Amelia Campbell, Jeremy Clark, Aisling Clifford, Laura Courtney, Ceri Dare, Kathleen Kelly, Chris Lynch, Paul McCrone, Shilpa Nairi, Karen Newbigging, Patrick Nyikavaranda, David Osborn, Karen Persaud, Martin Stefan, Brynmor Lloyd-Evans

**Affiliations:** 1 NIHR Mental Health Policy Research Unit, Division of Psychiatry, University College London, London, UK; 2 Camden and Islington NHS Foundation Trust, London, UK; 3 NIHR Mental Health Policy Research Unit, King’s College London, Institute of Psychiatry, Psychology & Neuroscience, London, UK; 4 NIHR Mental Health Policy Research Unit Co-Production Group, Division of Psychiatry, University College London, London, UK; 5 Mental Health Policy Branch, Department of Health and Social Care, London, UK; 6 Oxleas NHS Foundation Trust, London, UK; 7 Oxford Health NHS Foundation Trust, Oxford, UK; 8 Faculty of Education, Health and Human Sciences, University of Greenwich, London, UK; 9 Health Services Management Centre, School of Social Policy, University of Birmingham, Birmingham, UK; 10 Southern District Health Board, Southern Health, Dunedin, New Zealand

**Correction to: BMC Health Serv Res 21, 1174 (2021)**

**https://doi.org/10.1186/s12913-021-07181-x**

Following publication of the original article [1], the authors identified an error in the author name of Kathleen Kelly. The given name and family name were erroneously transposed.

The incorrect author name is: Kelly Kathleen.

The correct author name is: Kathleen Kelly.

In addition, an error was identified in the Discussion section.

The sentences currently read:

In addition to a lack of evidence, the criticism has been mainly focused on the ethics of police involvement in mental health care. The criticism has led to a coalition including people with relevant lived experience, clinicians and policy makers, protesting against the model’s deployment [49,50,51], resulting in policy makers requiring Trusts urgently to review its further use.

The sentences should read:

In addition to a lack of evidence, criticisms have included ethical problems with police involvement in mental health crisis care and potential harms of inappropriate diversion from healthcare. The StopSIM coalition of service users [49] has led a campaign against the model's deployment. This has led to concerns also being raised by professional and voluntary sector bodies [50-51], and the campaign has resulted in policy-makers requiring Trusts urgently to review the further use of SIM.

Reference 49 is changed to: StopSIM: Mental illness is not a crime. https://stopsim.co.uk/ Accessed 5/11/2021, and Reference 51 is changed with the previous Reference 49: Centre for Mental Health. Updated: Centre for Mental Health responds to the Serenity Integrated Mentoring (SIM) model. 2021.

The author group has been updated above and the original article [1] has been corrected.
