# Supplementary material for: Emerging models and trends in mental health crisis care in England: a national investigation of crisis care systems
Source: BMC Health Serv Res. 2021 Oct 29;21:1174. doi: 10.1186/s12913-021-07181-x (PMC8553397; doi:10.1186/s12913-021-07181-x)
Supplement: Supplementary file 2 — Additional file 2: Supplementary Table 1. Crisis assessment services. Supplementary Table 2. Crisis telephone services. Supplementary Table 3. Crisis houses. Supplementary Table 4. Crisis cafés. Supplementary Table 5. Acute day units (ADU). Supplementary Table 6. Police and/or ambulance street triage. Supplementary Table 7. Psychiatric decision units (PDU). Supplementary Table 8. Triage wards. [file 12913_2021_7181_MOESM2_ESM.pdf]

Emerging models and trends in mental health crisis care in England: a national investigation of crisis care systems

Supplementary Materials

Mental Health
Policy Research Unit
mhpru

Supplementary table 1: Crisis assessment services

	Yes		*No*		*Unsure/ Missing*
	n	*%*	*n*	*%*	*n*
Is there a separate crisis assessment service in your area?	71	38	114	62	15*
24hr	48	69	22	31	1**
Any member of public can self-refer	53	75	18	25	0**
Anyone known to the service can self-refer	59	83	12	17	0**
Can decide who should be treated by CRHTT without re-assessment	53	76	17	24	1**
Is there any shared staffing between the crisis assessment service and any other local crisis service?	35	50	35	50	1**
Is there any joint management between the crisis assessment service and any other local crisis service?	47	68	22	32	2**

*Number 'Unsure/Missing' out of all CRHTTs mapped (n=200). **Number 'Unsure/Missing' out of all areas with a crisis assessment service (n=71).

Mental Health Policy Research Unit mhpru

Supplementary table 2: Crisis telephone services

	Yes		*No*		*Unsure/ Missing*
	n	*%*	*n*	*%*	*n*
Is there a separate crisis telephone service in your area?	115	63	69	38	16*
Provider	-	-	-	-	2**
NHS	103	91	-	-	-
Voluntary	7	6	-	-	-
Local authority	3	3	-	-	-
Managed as part of another service	69	62	42	38	4**
24hr	83	73	31	27	1**
Any member of public can access	92	80	23	20	0**
Can decide who should be treated by CRHTT without re-assessment	30	26	85	74	0**
Can admit to hospital without reference to CRHTT	9	8	106	92	0**
Is there any shared staffing between the crisis telephone service and any other local crisis service?	34	32	73	68	8**
Is there any joint management between the crisis telephone service and any other local crisis service?	50	46	58	54	7**

*Number 'Unsure/Missing' out of all CRHTTs mapped (n=200). **Number 'Unsure/Missing' out of all areas with a crisis telephone service (n=115).

Supplementary table 3: Crisis houses

	Yes		No		Unsure/ Missing
	n	%	n	%	n
Is there at least one crisis house in your area?	85	47	95	53	20*
Is there > one crisis house in your area?	21	12	156	88	23*
The CRHTT has exclusive referral rights to a crisis house	46	26	134	75	20*
People in crisis can self-refer to a crisis house	14	8	166	93	20*
The CRHTT/ crisis assessment service can access a crisis house bed when needed?	-	-	-	-	2**
Always	25	30	-	-	-
Usually	49	59	-	-	-
Sometimes	7	8	-	-	-
Rarely	2	2	-	-	-
How quickly can admission to a crisis house be arranged?	-	-	-	-	3**
< 4 hours	35	43	-	-	-
< 24 hours	43	52	-	-	-
< 72 hours	4	5	-	-	-
Is there a local crisis house which is service user led?	6	8	73	92	6**
-	n	Median	IQR	-	Unsure/ Missing
Minimum age?***	80	18	0	-	5**
What is the typical length of stay, in days?***	70	7	5	-	15**
What is the maximum length of stay, in days?***	80	7	7	-	5**

*Number 'Unsure/Missing' out of all CRHTTs mapped (n=200). **Number 'Unsure/Missing' out of all areas with a crisis house (n=85). ***Calculated from the data regarding the first crisis house the participant told us about.

Supplementary table 4: Crisis cafés

	Yes		*No*		*Unsure/ Missing*
	n	***%***	***n***	***%***	***n***
Is there at least one crisis café in your area?	52	29	128	71	20*
Is there > one crisis café in your area?	12	7	159	93	29*
Is at least one crisis café provided by:	-	-	-	-	4**
Local authority	2	4	-	-	-
NHS	6	13	-	-	-
Voluntary sector	40	83	-	-	-
There is a crisis café open*	-	-	-	-	**4
Out of hours, Mon-Sun	32	67	-	-	-
Out of hours, Weekends	9	19	-	-	-
Out of hours, Weekdays	1	2	-	-	-
Office hours, Mon-Sun	2	4	-	-	-
Office hours, Weekends	0	0	-	-	-
Office hours, Weekdays	1	2	-	-	-
Other	3	6	-	-	-
Referral to crisis café	-	-	-	-	5**
Can drop in without appointment	32	68	-	-	-
Can self-referral but need to book an appointment	10	21	-	-	-
Referral needed from other health service	5	11	-	-	-
Is there a local crisis café which is service user led?	7	19	29	81	16**

*Number 'Unsure/Missing' out of all CRHTTs mapped (n=200). **Number 'Unsure/Missing' out of all areas with a crisis café (n=52). ***'Out of hours' = at least open 4 hours between 5pm and 9am; 'Office hours' = at least open 4 hours between 9am and 5pm; 'Weekends' = at least 2 evenings between Thu-Sun; 'Weekdays' = at least 4 days between Mon-Fri.

Mental Health Policy Research Unit mhpru

Supplementary table 5: Acute day units (ADU)

	Yes		No		Unsure/ Missing
	n	*%*	*n*	*%*	*n*
Is there at least one ADU in your area?	20	11	162	89	18*
Is there > one ADU in your area?	3	2	177	98	20*
The CRHTT has exclusive referral rights to an ADU	7	4	175	96	18*
People in crisis can self-refer to an ADU	1	1	181	99	18*
The CRHTT/ crisis assessment service can access an ADU place when needed?	-	-	-	-	0**
Always	8	40	-	-	-
Usually	11	55	-	-	-
Sometimes	1	5	-	-	-
Rarely	0	0	-	-	-
How quickly can admission to an ADU be arranged?	-	-	-	-	0**
< 4 hours	1	5	-	-	-
< 24 hours	11	55	-	-	-
< 72 hours	1	5	-	-	-
< 1 week	5	25	-	-	-
> 1 week	2	10	-	-	-
Is there a local ADU which is service user led?	1	5	18	95	1**
Is there any shared staffing between the ADU and any other local crisis service?	2	10	18	90	0**
Is there any joint management between the ADU and any other local crisis service?	6	32	13	68	1**
-	*n*	*Med-ian*	*IQR*	-	*Unsure/ Missing*
What is the typical length of time a patient can use this service, in days?	17	14	16	-	3**
What is the maximum length of time a patient can use this service, in days?	18	28	28	-	2**

*Number 'Unsure/Missing' out of all CRHTTs mapped (n=200). **Number 'Unsure/Missing' out of all areas with an ADU (n=20). ***Calculated from the data regarding the first ADU the participant told us about.

Supplementary table 6: Police and/or ambulance street triage

	Yes		No		Unsure/ Missing
	n	*%*	*n*	*%*	*n*
Is there a street triage in your area?	117	65	64	35	19*
Is it 24hr?	23	25	70	75	24**
What is the model of care	-	-	-	-	8**
Mobile unit with mental health worker and police staff	44	40	-	-	-
Mental health worker based in service call centre – can attend calls	23	21	-	-	-
Mental health worker based in service call centre – phone advice only	19	17	-	-	-
Other	23	21	-	-	-
Is there any shared staffing between the police street triage and any other local crisis service?	46	46	64	64	7**
Is there any joint management between the police street triage and any other local crisis service?	61	55	49	45	7**

*Number 'Unsure/Missing' out of all CRHTTs mapped (n=200). **Number 'Unsure/Missing' out of all areas with a police street triage (n=116).

Supplementary table 7: Psychiatric decision units (PDU)

A PDU is a dedicated area (separate to an A&E department / psychiatric ward / psychiatric liaison team) in which assessment can be conducted and treatment plans developed for patients in mental health crisis who are accessing emergency services. People may typically stay for up to 24-48 hours in a PDU, which may be called a "Clinical Decision Unit" or other in some areas.

	Yes		No		Unsure/ Missing
	n	*%*	*n*	*%*	*n*
Is there a PDU in your area?	30	16	152	84	18*
Where is the PDU located	-	-	-	-	0**
Psychiatric hospital	21	70	-	-	-
Acute hospital	6	20	-	-	-
Other	3	10	-	-	-
Is the PDU co-located with any other service?	14	50	14	50	2**
Is it 24hr?	27	96	1	4	2**
What is the accommodation on the PDU like?	-	-	-	-	2**
Single room/ Partitioned area	3	11	-	-	-
Beds	1	4	-	-	-
Recliners	23	82	-	-	-
No overnight accommodation	0	0	-	-	-
Other	1	4	-	-	-
The CRHTT or crisis assessment service can refer	20	69	9	31	1**
The psychiatric liaison service can refer	26	90	3	10	1**
When can the PDU admit to hospital?	-	-	-	-	1**
Without CRHTT or crisis assessment service input	12	44	-	-	-
Following phone discussion with CRHTT / crisis assessment service	8	30	-	-	-
Only following in person assessment of the patient by CRHTT / crisis assessment service	7	26	-	-	-
Other	2	-	-	-	-
Is there any shared staffing between the PDU and any other local crisis service?	21	75	7	25	2**
Is there any joint management between the PDU and any other local crisis service?	19	70	8	30	3**
-	*n*	*Median*	*IQR*	-	*Unsure/ Missing*
How long is the maximum length of stay, in hours?	25	23	25	-	5**
What is the capacity of the PDU?	23	6	1.5	-	7**

*Number 'Unsure/Missing' out of all CRHTTs mapped (n=200). **Number 'Unsure/Missing' out of all areas with a PDU (n=30).

Supplementary table 8: Triage wards

An inpatient psychiatric ward which only accepts admissions for a time-limited period (not more than one week); typically does not admit people compulsorily detained under a MHA section; and works closely with local community crisis services to avoid the need to transfer to an acute psychiatric ward.

	Yes		No		*Unsure/ Missing*
	n	*%*	*n*	*%*	*n*
Is there a triage ward in your area?	7	4	167	96	26*
The CRHTT or crisis assessment service can refer	6	86	1	14	0**
The psychiatric liaison service can refer	5	71	2	29	0**
When can the triage ward admit to hospital?	-	-	-	-	0**
Without CRHTT or crisis assessment service input	3	75	-	-	-
Following phone discussion with CRHTT / crisis assessment service	1	25	-	-	-
Only following in person assessment of the patient by CRHTT / crisis assessment service	0	0	-	-	-
Other	3	-	-	-	-
Is there any shared staffing between the triage ward and any other local crisis service?	1	17	5	83	1**
Is there any joint management between the triage ward and any other local crisis service?	0	0	6	100	1**
-	*n*	*Median*	*IQR*	-	*Unsure/ Missing*
How long is the maximum length of stay, in hours?	5	72	0	-	2**
How many beds does it have?	5	18	2	-	2**

*Number 'Unsure/Missing' out of all CRHTTs mapped (n=200). **Number 'Unsure/Missing' out of all areas with a triage ward (n=7).

Mental Health Policy Research Unit mhpru
