# Supplementary material for: Emerging models and trends in mental health crisis care in England: a national investigation of crisis care systems
Source: BMC Health Serv Res. 2021 Oct 29;21:1174. doi: 10.1186/s12913-021-07181-x (PMC8553397; doi:10.1186/s12913-021-07181-x)
Supplement: Supplementary file 3 — Additional file 3. Participant Information Sheet. [file 12913_2021_7181_MOESM3_ESM.pdf]

INFORMATION SHEET FOR NATIONAL SURVEY OF MENTAL HEALTH CRISIS SERVICES IN ENGLAND

A service evaluation commissioned by the Department of Health and Social Care (DHSC)

You are being invited to participate in a national survey of mental health crisis care systems. Please take time to read the following information carefully and discuss it with others if you wish. Ask us if there is anything that is not clear or if you would like more information.

What is the purpose of this survey?

The purpose of this survey is to identify local services for people who are experiencing a mental health crisis how they work together and how they can be accessed by people in crisis. This will allow us to describe local acute care systems. The NHS Long Term Plan makes a commitment to increase investment in crisis pathways and there is strong support nationally and in many local services for integrated crisis pathways with a range of options that meet different needs and preferences. However, the evidence base regarding effective crisis systems limited. This survey will support national and local investment decisions over the next 5 years and inform an ongoing programme of research commissioned by the DHSC. This survey aims to complement the annual NHS England survey of mental health crisis teams, and has been developed with input from NHS England (NHSE).

Who is carrying out the survey?

The DHSC has commissioned the National Institute for Health Research Mental Health Policy Research Unit (MHPRU) to conduct the survey. The team is based at University College London and led by Professor Sonia Johnson and Dr Brynmor Lloyd-Evans.

Why have I been asked to take part?

We are contacting the manager or a senior clinician of all Crisis Resolution and Home Treatment Teams (CRHTTs), Crisis assessment teams or equivalent services in England to invite them to participate in the survey. This includes crisis services for children and young people and older adults. If there are no CRHTTs or equivalent services in the Trust where you work, we have asked you as a manager or senior clinician from another NHS crisis service. We may contact additional senior managers or clinicians within the Trust with good knowledge of local crisis services to obtain any missing information or to check reliability of data in a sample of survey responses.

Do I have to take part?

You are not obliged to participate in this survey and deciding not to participate will have no negative effects on you or on your service. However, the survey is supported by both the DHSC and NHSE, so your assistance with this national policy project would be greatly appreciated. A letter of support for this survey from DHSC can be provided by the research team.

What does this survey involve?

This survey will involve completing a questionnaire about your service and other local crisis services you often work with. We will ask about what local crisis services are available, referral and care pathways between services, and how they are integrated into the local crisis care system. We recommend completing the survey as a telephone interview with a study researcher, or you can

complete the form online. The survey should take about 30 minutes to complete, depending on the complexity of local crisis service provision.

What are the possible benefits of my participation?
The survey will support the work of the DHSC and NHSE in implementing the NHS Five Year Forward View and Long Term Plan and evaluating the effectiveness of the Beyond Places of Safety programme. The potential benefit for the participants will come from information being made available to inform optimal local systems of crisis care.

What are the possible disadvantages of my participation?
The main disadvantage is that we are aware staff and managers in mental health services experience great pressure on their time. Participating in this survey will take about 30 minutes of your time. Otherwise we doubt you will find participation in the survey distressing in any way.

Confidentiality and Consent
A summary of the findings will be written up in a report for the DHSC and NHSE. In this report, and in any other publications resulting from this survey, the identity of survey respondents will be anonymised. We will provide the data from the survey to the DHSC and NHSE at the end of the survey: we will remove respondents' names from this data set so you will not be personally identified, but the service in which you work and other local crisis services will be identifiable. As this survey meets criteria for a service evaluation rather than a research study, we will not ask you to complete and sign a consent form but the study researcher will check you have understood the information provided here and are happy to participate before you start the survey as a telephone interview. If you choose to complete the survey online, the survey includes an initial button to check to confirm you have understood this information and agree to take part.

Where can I get further information?
If you require any further information or have any questions not answered by this information sheet, or if you have any comments or concerns, please do not hesitate to contact a member of the research team. The research team representatives could be the researcher who is conducting the interview or the study leads:

Professor Sonia Johnson
Division of Psychiatry, University College London
Email: s.johnson@ucl.ac.uk (Telephone) 020 7685 5757

Dr Brynmor Lloyd-Evans
Division of Psychiatry, University College London
Email: b.lloyd-evans@ucl.ac.uk (Telephone) 020 7679 9428

What if I am unhappy with the survey?
If you have any concerns about the survey or how you have been treated during its course, the researcher will be very happy to discuss this with you. You could also contact the Study Leads, whose contact details are above. If you wish to complain formally, or have any unresolved concerns about any aspect of the way you have been approached or treated during the course of this survey, the normal National Health Service complaints mechanisms are available to you.

What happens to the results of the survey?
The completed questionnaires will be stored securely using Opinio, accessible only to the research team. The information collected will be written up in a report for the DHSC. Your name will be removed from this report but the name of your service will be included. Anonymised results may also be

published in scientific journals and publications read by mental health service clinicians and service users. We will send you a full report on the overall study findings once our survey is complete.

Who is organising and funding the survey?
The survey is commissioned and funded by the DHSC.

Who has reviewed the survey?
The survey has been reviewed by the North London Research Consortium (NoCLOR) and meets Health Research Authority criteria for service evaluation rather than research. We have informed senior managers in each NHS Trust that this survey is taking place, and will comply with any local approval processes as directed.

Thank you for reading this information sheet
